# Supplementary material for: Antennal Sensilla Morphology and Flagellomere Addition in Nymphs and Adults of Hierodula patellifera Serville, 1839 (Mantodea: Mantidae)
Source: Insects. 2025 Jun 24;16(7):655. doi: 10.3390/insects16070655 (PMC12295877; doi:10.3390/insects16070655)
Supplement: Supplementary file 1 [file insects-16-00655-s001.zip › insects-3617144-supplementary.pdf]

Table S1. Length (μm) and width (μm) of antennal segments of nymphs and adults of *H. patellifera.*

Instar	Scape		Pedicel		Length of antennae
	Length	Width	Length	Width	
1st instar	197.2±21.8^{i}	153.1±31.6^{h}	152.5±18.6^{i}	106.8±7.2^{i}	5873.3±315.5^{m}
2nd instar	226.3±12.1^{h}	167.8±8.9^{g}	168.5±8.4^{h}	112.9±6.6^{h}	6808.6±527^{l}
3rd instar	232.9±15.3^{g}	186.8±11.6^{f}	195.3±22.9^{g}	125.9±8.6^{g}	7773±339.6^{k}
4th instar	237.1±8.5^{g}	190.9±16.2^{f}	202±21.4^{f}	146.9±12.2^{f}	8489.1±374.7^{j}
5th instar	265.8±7.6^{f}	230.9±11.8^{e}	239.2±11.7^{e}	176.8±14.3^{e}	9816.2±507^{i}
6th instar	355.7±13.2^{e}	303.2±12.5^{d}	286.7±36.4^{d}	197.8±18^{d}	12211.7±565.7^{h}
7th female	385.8±16.4^{d}	313.4±23.5^{d}	316.7±33.9^{c}	212.4±27.7^{c}	14292.7±343.4^{g}
7th male	397.3±20.1^{d}	323.7±25.7^{d}	320.9±30.3^{c}	218.9±24.34^{c}	15301.6±358.9^{f}
8th female	445.3±30.9^{c}	347.2±35.6^{c}	326.7±31.4^{c}	227.7±46.8^{b}	17922.7±278.9^{e}
8th male	467.3±39.1^{c}	353.2±30.6^{c}	329.5±37.9^{c}	231.4±11.3^{b}	18685.9±400.2^{d}
9th female	479.3±32.5^{b}	370.2±31.8^{b}	344.8±30.5^{b}	241.7±32.6^{b}	19853.3±686.4^{c}
9th male	482.3±30.1^{b}	382.5±29.7^{b}	352.9±32.4^{b}	251.8±29.1^{b}	20996±676.2^{b}
Adult female	521.3±30.8^{a}	460.3±29.6^{a}	371.7±23.3^{a}	277.7±46.8^{a}	17806.7±564.3^{e}
Adult male	538.3±30.6^{a}	480.9±42.4^{a}	411.9±31.2^{a}	310.4±22.7^{a}	21432.9±552.1^{a}

Each column of data is analyzed together, and the letters in the table indicate significant differences ($p < 0.05$, Tukey). Notes: Letters with the same mark indicate no significant difference, while letters with different marks indicate a significant difference.

 https://doi.org/10.3390/insects16070655

Table S2. The length and width of sensilla in nymph and adult *H. patellifera* antennae.

Instar	Length					Width				
	slender Sc	mid-length Sc	robust Sc	StI	StII	Slender Sc	mid-length Sc	Robust Sc	StI	StII
1st instar	48.13-54.9	32.44-37.44	/	32.44-39.76	20.59-24.15	2.7-2.81	2.2-2.59	/	1.84-1.91	1.94-1.98
2nd instar	53.9-59.33	35.3-38.12	/	33.19-40.46	22.98-24.87	2.72-2.94	2.47-3.09	/	1.94-1.95	1.98-1.99
3th instar	55.5-61.31	37.5-38.65	/	34.7-40.85	23.06-25.27	3.08-3.15	2.76-3.23	/	1.97-1.99	1.99-2.01
4th instar	55.63-63.57	37.65-38.83	22.99-33.05	35.93-41	23.22-25.39	3.62-3.73	2.78-3.32	3.26-3.73	1.98-2	2-2.02
5th instar	57.35-67.12	38.68-42.45	25.56-37.75	36.94-41.21	23.26-25.76	3.86-3.95	2.84-3.52	3.32-3.92	1.99-2.14	2.12-2.14
6th instar	72.26-83.58	44.38-48.78	25.77-39.23	37.1-42.02	23.34-25.86	4.12-4.47	3.4-4.23	3.38-4.43	2.09-2.28	2.13-2.36
7th female instar	84.19-98.11	48.62-55.32	26.88-40.12	37.86-43.9	24.52-26.7	4.36-5.1	3.78-4.82	3.42-4.55	2.11-2.3	2.14-2.38
7th male instar	89.4-108.84	56.38-60.05	27.85-44.67	37.22-45.94	25.25-28.18	4.33-5.13	3.87-4.88	3.53-4.65	2.12-2.34	2.15-2.44
8th female instar	90.9-106.27	63.35-67.9	27.65-42.83	38.11-46.3	24.96-29.22	4.86-5.25	3.82-5.21	3.6-4.93	2.13-2.39	2.15-2.52
8th male instar	97.38-114.76	62.73-71.32	28.63-45.13	38.56-46.12	25.75-28.72	4.9-5.2	3.89-5.27	3.62-4.97	2.15-2.4	2.16-2.57
9th female instar	101.3-110.2	60.4-76.94	28.77-45.45	39.23-47.29	25.11-29.8	4.94~5.38	3.91-5.5	3.53-5.32	2.15-2.48	2.26-2.69
9th male instar	107.67-118.3	65.54-78.93	29.11-46.23	39.78-47.87	25.62-29.12	4.9~5.4	3.93-5.72	3.76-5.34	2.16-2.57	2.29-2.75
adult female	105.26-115.33	64.52-80.5	32.12-52.71	41.16-49.23	27.7-29.48	5.12-5.51	5.61-5.87	3.81-6.21	2.22-2.65	2.3-3.18
adult male	108.3-125.05	65.73-81.72	28.75-51.96	41.32-50.4	27.3-29.61	5.35-6.34	5.96-6.36	3.67-6.46	2.32-2.7	2.5-3.33
